# Functional Genomics of PRUNE1 in Neurodevelopmental Disorders (NDDs) Tied to Medulloblastoma (MB) and Other Tumors

**DOI:** 10.3389/fonc.2021.758146

**Published:** 2021-10-22

**Authors:** Francesca Bibbò, Carmen Sorice, Veronica Ferrucci, Massimo Zollo

**Affiliations:** ^1^ Dipartimento di Medicina Molecolare e Biotecnologie Mediche (DMMBM), ‘Federico II’ University of Naples, Naples, Italy; ^2^ CEINGE Biotecnologie Avanzate, Naples, Italy

**Keywords:** PRUNE_1, medulloblastoma, metastasis, proliferation rate, microtubules polymerization, Wnt, TGF—transforming growth factor, neurodevelopmental disorder with microcephaly, hypotonia, variable brain anomalies (NMIHBA)

## Abstract

We analyze the fundamental functions of Prune_1 in brain pathophysiology. We discuss the importance and maintenance of the function of Prune_1 and how its perturbation influences both brain pathological conditions, neurodevelopmental disorder with microcephaly, hypotonia, and variable brain anomalies (NMIHBA; OMIM: 617481), and tumorigenesis of medulloblastoma (MB) with functional correlations to other tumors. A therapeutic view underlying recent discoveries identified small molecules and cell penetrating peptides to impair the interaction of Prune_1 with protein partners (e.g., Nm23-H1), thus further impairing intracellular and extracellular signaling (i.e., canonical Wnt and TGF-β pathways). Identifying the mechanism of action of Prune_1 as responsible for neurodevelopmental disorders (NDDs), we have recognized other genes which are found overexpressed in brain tumors (e.g., MB) with functional implications in neurodevelopmental processes, as mainly linked to changes in mitotic cell cycle processes. Thus, with Prune_1 being a significant target in NDDs, we discuss how its network of action can be dysregulated during brain development, thus generating cancer and metastatic dissemination.

## Introduction

*PRUNE_1*, the human homolog of the *Drosophila melanogaster pn* gene, was firstly identified by viable mutations responsible for the brunish purple eye color compared to the red of wild-type fly (1). The phenotype of *pn* mutant flies was due to alterations in the metabolism of drosopteridines (1). Interestingly, the same homozygous or hemizygous *pn* mutation became lethal when the protein encoded by abnormal wing disc (i.e., *awd*) gene was also mutated (i.e., p.P97S) (2). *Awd* is the homolog of the human *NME1* gene encoding for the Nm23-H1 (or NDPK-A) protein, one of the known Prune_1 binders. In this regard, the double mutant flies died at the second/third larval developmental stage because of anomalies of mesoderm development and central nervous system (CNS)-like cells. Interestingly, the mutant p.P97S *awd* protein still retained the ability to bind Prune_1 protein (3). Thus, the “lethal interaction” between *awd* and *pn* mutant proteins was hypothesized to be due to neomorphic mutations unbalancing the switch from guanosine triphosphate (GTP) to guanosine diphosphate (GDP; mainly mediated by *awd* protein), thus causing alterations in the modulation of GTPases and/or GTPase-activating proteins (4).

Importantly, Prune-M1 and Nm23-M1 (the mouse homologs of the human *PRUNE_1* and *NME1* genes, respectively) showed significant co-expression patterns in the cortex, hippocampus, midbrain, and the cerebellum during murine neural development, thus suggesting a critical role for these proteins during neural development (5).

The human Prune_1 protein belongs to the DHH (Asp-His-His) protein superfamily (that also includes the *pn* protein of *Drosophila* and the RecJ exonuclease of bacteria) and possesses a nucleotide phosphodiesterase (PDE) (6) and an exopolyphosphatase (exopolyphosphatase/pyrophosphatase, PPX/PPase) action, with greater affinity for short-chain over long-chain inorganic polyphosphates (polyPs) (7). Regarding its tertiary structure, human Prune_1 is a naturally unfolded protein with the ability to interact with several intracellular binding partners, including GSK-3β (8) and Nm23-H1/H2 (9). Furthermore, Prune_1 was also identified as a microtubule-associated protein (MAP) with a role in the enhancement of microtubule (MT) polymerization in the mitotic spindle during cell division (10).

Because of the enzymatic activities and its interaction with several proteins, Prune_1 was found able to modulate both intra- and extracellular signaling cascades [including the canonical Wnt (8) and TGF-β (11) pathways] that regulate cell proliferation, motility, and epithelial–mesenchymal transition (EMT) processes.

To date, Prune_1 has been found highly expressed and positively correlated with the grading, EMT, and metastatic status in several tumors, including those of the CNS and the peripheral nervous system (PNS), and in medulloblastoma (MB) and neuroblastoma (NBL). Indeed, high expression levels of Prune_1 were found in metastatic MB (11), the most common childhood brain tumor. In MB group 3, Prune_1 drives a “metastatic axis,” thus leading to the enhancement of the TGF-β pathway, activation of EMT process, and the reduction of the amount of PTEN protein (11).

Furthermore, recessive mutations in *PRUNE_1* locus (1q21.3) were also identified as candidate genetic causes of neurodevelopmental disorder with microcephaly, hypotonia, and variable brain anomalies (NMIHBA; MIM #617481) (10, 12–20). Similarly, other genes responsible for microcephaly (MCPH) and mainly implicated in cell division mechanisms were also found both overexpressed in brain tumors (i.e., MB) and mutated in neurodevelopmental disorders (NDDs).

In this review, we will focus on the Prune_1 protein enzymatic activities, intracellular pathways, and protein interactors in the interplay between metastatic CSN tumor (i.e., MB) and NDD (i.e., NMIHBA) pathogenesis. In conclusion, we also highlight the use of Prune_1-inhibitors as new potential therapies for the treatment of brain tumors. In the near future, once the mechanisms have been identified, we will then use this knowledge to understand their use and potential application in therapeutic interventions also in NDDs.

## Prune_1 Enzymatic Activities

Prune_1 protein is part of DHH phosphoesterase superfamily. Its N-terminal domain contains the DHH motifs (6). Prune_1 retains two different enzymatic functions—PDE (6) and PPX/PPase activities—the latest with an order of magnitude higher (7).

### Phosphodiesterase Activity

Regarding PDE activity, Prune_1 has the ability to hydrolyze the second messengers adenosine and guanosine 3′,5′-cyclic nucleotides [i.e., cyclic adenosine monophosphate (cAMP) and cyclic guanosine monophosphate (cGMP)] in their corresponding 5′-monophosphates (i.e., 5′-AMP and 5′-GMP, respectively). cAMP has major affinity to Prune_1, then cGMP (*K*_m_ values: cAMP, 0.9 ± 0.03 M; cGMP, 2.3 ± 0.11 M) (6). Phosphodiesterase enzymes have important roles in cellular homeostasis because of the pivotal functions of cAMP and cGMP (as second messengers) in physiological processes due to the modulation of signaling pathway transduction (21). Furthermore, Prune_1 PDE activity has been reported to enhance cell motility *in vitro* (6). In this regard, MBA-MB-435 cells that overexpressed mutant Prune_1 proteins lacking PDE activity [through amino acid changes within the region containing motif III (DHRP126-129AAAA, PruneΔ) were found with decreased migration rates (6). Furthermore, dipyridamole, a PDE inhibitor, was also found to affect the motility of breast cancer (BC) cells (i.e., MDA-MB231T) (22, 23). These data, thus, suggest that the PDE enzymatic activity of Prune_1 protein may have a role in the modulation of the migratory properties of tumorigenic cells in which Prune_1 is overexpressed.

### Exopolyphosphatase (PPX/PPase) Activity

The PPX/PPase (polyphosphate–phosphohydrolases) activity of Prune_1 was found to exceed its PDE function (7). Prune_1 has affinity for short-chain over long-chain inorganic polyPs. Indeed, it can hydrolyze linear polyPs by using Mg^2+^ or Co^2+^ as a cofactor. Among the inorganic polyPs with different chain lengths, the highest *K*_cat_ values for Prune_1 were observed by using triphosphates (P3), adenosine 5′-tetraphosphate (AP4), and guanosine 5′-tetraphosphate (GP4) as substrates. These values dramatically decreased with polyPs at increased chain lengths. Interestingly, PPi, ATP, and GTP were not significantly hydrolyzed (7). Of importance is that loss of PPX/PPase activity was found in the recombinant mutants p.N24H, p.D28A, p.D179A, and p.R348A Prune_1 proteins when assayed in the presence of P3 and Mg^2+^ as the substrate and cofactor, respectively. The other mutants, p.D106A, p.H107N, and p.H108N Prune_1 proteins, had reduced but measurable PPX/PPase activity. In contrast, the mutant p.R128H Prune_1 protein showed an increased *K*_cat_ value in comparison with the wild-type protein (7). These results suggest that variations within the DHH domain (e.g., p.D106A, p.H107N, and p.H108N) did not affect the PPX/PPase activity of Prune_1 (7). In contrast, the same PPX/PPase enzymatic function resulted affected in mutant Prune_1 proteins containing variations in those residues conserved in PPase and PPX enzymes (i.e., p.D28A, p.D179A, and p.R348A) (7).

At this time, questions were raised about the potential role of PPX/PPase activity in cell motility. In this regard, a delayed migratory rate was shown in HaCaT cells overexpressing PPX1 (the *Saccharomyces cerevisiae* homolog of the human *PRUNE_1* gene) by performing would healing assays (24). However, different functions were also postulated due to the role that long-chain polyPs play in the induction of ERK1-2 (i.e., MAP kinase pathway) in human cells (25). Thus, how the PPX/PPAse activity of Prune_1 influences migratory processes and cell motility in both physiological conditions is still not fully understood (in the context of polyP degradation and/or organelle cellular compartment storage). Future research studies and discoveries will address these questions.

Inorganic polyPs are polymers of linear orthophosphate (Pi) units ranging from five and several thousand orthophosphates that are linked through phosphoanhydride bonds (one of the most energy-rich linkages). In human, their amounts are very high in blood plasma, platelets, and osteoblasts, where they modulate blood clotting (26), mineralization processes, and the regulation of ATP level (27). Thus, polyPs are involved in many functions mostly related to additional sources of energy into the cells. Interestingly, polyPs were found to act as cytoprotective agents against human immunodeficiency virus type 1 (HIV-1) (28). In this regard, very recently, polyPs have been found able to exert antiviral actions against severe acute respiratory syndrome coronavirus 2 (SARS-CoV-2) (29), during early phase of infection and replication (30).

Furthermore, high concentrations of polyPs were also found in mammalian brain, especially in astrocytes, where they were found to induce calcium signaling through P2Y1 purinergic receptors and phospholipase C induction, inositol 3 phosphate (IP3) delivery, and cytosolic Ca^2+^ increase (31, 32). Moreover, PolyPs were also reported to amplify the pro-inflammatory response in endothelial cells *via* the activation of the same P2Y1 receptor (33), thus overall modulating mitochondrial functions. In this regard, the depletion of mitochondrial polyPs (through the overexpression and the activity of yeast PPX) significantly increased the levels of orthophosphates in the mitochondrial matrix (which is necessary for Ca^2+^ binding), thus resulting in a decreased mitochondrial membrane potential and the inhibition of complex I of the respiratory chain (34, 35). Thus, the crucial role of polyPs in cell metabolism is clear, as also confirmed in transgenic mice widely expressing the scPPX1 exopolyphosphatase that displayed a reduced mitochondrial respiration in muscles (36).

## Prune_1 Protein Interaction With Protein Binding Partners

Human Prune_1 is a naturally unfolded multi-domain adaptor protein. The three-dimensional structure of the carboxyl-terminus (C-terminus) region of Prune_1 was obtained through nuclear magnetic resonance (NMR) studies and molecular dynamics techniques (37). Two globular regions responsible for its enzymatic activities (i.e., DHH and DHHA2 domains) were found within the amino-terminus (N-terminus) domain of Prune_1 (37). The DHHA2 domain also contained a conserved DXK motif at its N-terminus. An intrinsically disordered domain (starting at 371 amino acid residue) containing two stretches with a tendency for helix structures was also identified. The C-terminal domain of Prune_1 is characterized by residues (postulated to reside between 393 and 420) that are involved in its homodimerization and a small globular region responsible for its interactions with other proteins (38, 39). In fact, Prune_1 was found with the ability to bind several intracellular protein partners mainly involved in cytoskeletal rearrangement, including α- and β-tubulins, glycogen synthase kinase 3β (GSK-3β) (40), and Nm23-H1/H2 (or NDPK-A/B) (6). Through these interactions, Prune_1 can modulate different intracellular pathways that might be then also translated into extracellular signaling, including the canonical Wnt *via* GSK-3β (8) and TGF-β *via* Nm23-H1 binding (11).

### Prune_1 Interaction With GSK-β

The region of Prune_1 responsible for the interaction with GSK-3β starts from 330 to 394 amino acid residues within the C-terminus domain (8, 40). Because of the co-localization between Prune_1 and the focal adhesion proteins (i.e., F-actin, paxillin, and vinculin) in several cell types, this Prune_1–GSK-3β interaction suggests that Prune_1 can also modulate the microtubular architecture and dynamics (40–42). Importantly, GSK-3β is considered a negative modulator of the canonical WNT/β-catenin pathway through phosphorylation at its amino acid residues S9 and S21 (8, 9). Of importance is that, through GSK-3β interaction, Prune_1 was found to activate β-catenin signaling cascade and to further promote Wnt3a secretion in non-small cell lung cancer (NSCLC) cells (8); thus, a connectome related to the WNT signaling activation was identified (43).

### Prune_1 Interaction With NME-1 (Nm23-H1)

Prune_1 was also shown to physically interact with the C-terminus domain of Nm23-H1 through its D388 and D422 residues (37). *Nm23-H1* is described as the first anti-metastatic gene with different functions: a nucleoside diphosphate kinase (NDPK) activity that catalyzes the transfer of a phosphoryl group from a nucleoside triphosphate (NTP) to a nucleotide diphosphate (NDP), a histidine kinase function, and a 3′−5′ exonuclease activity (44). In detail, *in vitro* studies demonstrated that the complex formation involved the dimer of Prune_1 and the hexameric form of Nm23-H1 (38). Interestingly, Nm23-H1 and GSK-3β could bind Prune_1 at the same time by using non-identical regions of its C-terminus domain for their binding, thus suggesting two independent interaction sites for signaling complex assembly (38). Moreover, other *in vitro* studies demonstrated that the Prune_1/Nm23-H1 interaction complex requires casein kinase I/II-mediated phosphorylation on three serine residues (S120, S122, and S125) of Nm23-H1. Phosphorylated Nm23-H1 on S120, S122, and S125 was also found to increase its hexameric form (41). Of importance the overexpression of both Prune_1 and Nm23-H1 was found in different tumors, such as MB (11), NBL (37) and BC (45). In this regard, the data indicate that the Prune_1/Nm23-H1 complex could affect the anti-metastatic activity of Nm23-H1 and increase the cell motility properties (45, 46).

### Prune_1 Interaction With α- and β-Tubulins

Other potential Prune_1 interactors were identified through a pull-down assay coupled to mass spectrometry analysis in BC cells (42). Through this approach, new potential Prune_1 binders were identified. Among these, the interaction between Prune_1 and β-tubulin was proposed because of the crucial functions played by tubulins, MTs, and MAPs in neural development (47, 48). Furthermore, this interaction was also confirmed by co-immunoprecipitation assays (Prune_1 protein and both β- and α-tubulins) in NBL inducible cell clones (SH-SY5Y) (10). Of significance is that Prune_1 protein was also identified as a MAP by performing a cell-based microtubule-binding protein spin-down assay using the same neuronal SH-SY5Y cell clones (10). These data were also confirmed by MT polymerization assays *in vitro* and “in cells,” thus showing an enhancement of the MT polymerization rate (nucleation, growth, and steady-state phases) in the presence of a recombinant wild-type Prune_1 protein (10). Furthermore, Prune_1 was also found to co-localize with MTs (i.e., β-tubulin) in mitotic spindles during cell division (prometaphase, metaphase, anaphase, and cytokinesis) in Hela cells (10). Taken altogether, these data indicate an important role for Prune_1 protein in cellular division processes.

## Prune_1 in Neurodevelopmental Disorder

### The Role of Prune_1 During Neural Development

Prune_1 was found to take part in fundamental processes occurring during neural development. In this regard, Prune-M1 and Nm23-M1 (i.e., the murine homologs of human *PRUNE_1* and *NME-1*, respectively) had similar expression patterns in the developing murine nervous system, from early brain development to adulthood (5). In fact, a significant spatiotemporal co-expression of both Prune-M1 and Nm23-M1 during brain development, within the cortex, hippocampus, and midbrain, was observed. Regarding the telencephalon, both genes were found expressed in the ventricular zone (VZ) of the neopallial cortex, a region involved in proliferation processes. Furthermore, the expressions of Prune-M1 and Nm23-M1 were also shown within the intermediate zone and in the ganglionic eminence. These regions allow neurons to migrate to the pial surface of the forebrain to then undergo differentiation processes. Furthermore, Prune-M1 and Nm23-M1 were also expressed in the cerebellum during development in both granular and Purkinje neuron precursors (5). These findings suggest fundamental functions for Prune_1 and Nm23-H1 during cell proliferation, migration, and differentiation processes occurring in the developing brain and mainly in the cerebellum. Of interest is that the expressions of Prune-M1 and Nm23-M1 were also shown during the murine postnatal phases in cortical layers II and IV and in the basal ganglia of the striatum, thus indicating a potential involvement of the complex in somatosensory processing and in synaptic function and plasticity (5). Interestingly, Nme-X1 and Prune-X1 (i.e., *Xenopus* homolog proteins) were also reported with a potential function in Muller gliogenesis during retinal development in *Xenopus* (49). This, thus, indicates a role for the Prune_1 and Nm23-H1 complex also during the development of the retina and in eye morphogenesis.

### Recessive Mutations in *PRUNE_1* Locus Are Responsible for NDD

Recently, homozygous and composite heterozygous mutations in *PRUNE_1* locus (1q21.3) were found [through whole-exome sequencing (WES) analyses] in several patients worldwide as causative of the autosomal-recessive NMIHBA (MIM #617481). NMIHBA is a severe disease due to the global developmental delay and the intellectual disability. In fact, most of the affected patients presented MCPH and brain, cerebellar, and optic atrophy. Other cerebral abnormalities were also reported in these patients (through brain imaging), such as cortical atrophy, thickness of the corpus callosum, and hypoplasia of the cerebellum. Some patients were also described with seizure, peripheral spasticity, and slight delay in myelination process. Furthermore, the phenotype is often accompanied by a decreased muscle tone (i.e., “truncal hypotonia”), impossibility to ambulate, or speech and broad dysmorphism.

**Figure 1:**
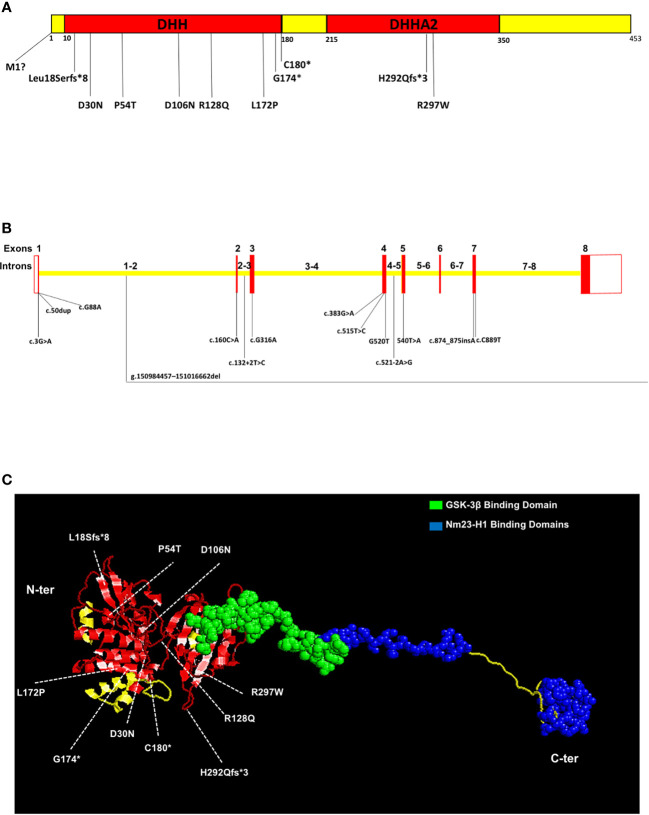
A schematic representation of the Prune_1 protein and *PRUNE_1* gene with the mutations and binding regions, as reported in the literature. **(A)** Prune_1 protein is composed of 453 amino acids harboring the DHH (Asp-His-His) (from amino acid residues 10 to 180) and DHHA2 (from residues 215 to 350) domains. In the DHH domain, the truncating variant p.L18Sfs*8, the missense mutations p.M1?, p.D30N, p.P54T, p.D106N, p.R128Q, p.L172P, and the composite heterozygous mutations p.R128Q;G174X, and p.D106N;C180* have been identified. In the DHHA2 domain, the missense variant p.R297W and the homozygous frameshift variant p.H292Qfs*3 have been identified. **(B)** Exons are denoted by *red boxes*, UTRs are denoted by *white boxes*, and introns are denoted by *yellow boxes*. In exon 1 are shown the homozygous truncating c.50dup variant and the homozygous mutation c.G88A. The homozygous deletion g.1509-84457–151016-662del involves exons 2–8. Other homozygous mutations are found in exon 1 (c.3G>A) exon 2 (c.160C>A), exon 3 (c.G316A), and exon 4 (c.383G>A, c.515T>C, and G520T). The mutation c.132+2T>C was found in the splice donor site within intron 2, while the c.521-2A>G variant was found in the canonical splice acceptor site in intron 4. The homozygous frameshift variant c.874_875insA and the missense mutation c.C889T were found in exon 7. **(C)** Three-dimensional representation of Prune_1 protein from the N-terminal (*left*) to the C-terminal (*right*). The DHH (from amino acid residues 10 to 180) and DHH2 (from residues 215 to 350) domains are shown in *red*. The GSK-3β binding domain (from residues 330 to 394) is represented in *green*. The Nm23-H1 binding domains (from amino acid residues 388 to 402 and from 422 to 446) are depicted in *blue*. We additionally included in both domains of the protein information about the mutations published in the literature. In the DHH domain the truncating variant p.L18Sfs*8, the missense mutations p.D30N, p.P54T, p.D106N, p.R128Q, and p.L172P, and the composite heterozygous mutations p.R128Q;G174X and p.D106N;C180* have been represented. In the DHHA2 domain, the missense variant p.R297W and the homozygous frameshift variant p.H292Qfs*3 have been represented.

This complex phenotypic spectrum in NMIHBA patients can be reasoned by the genotypic diversity in *PRUNE_1* locus. To date, 64 patients carrying different mutations in the *PRUNE_1* gene have been reported worldwide (**Figure 1** and **Table 1**). Regarding the genotypic differences, among the variants identified in patients with *PRUNE_1*, the most representative was the homozygous c.G316A (p.D106N) variant that was found in 15 subjects: seven from Turkey (12, 16, 18), three from Italy (10, 14), one from Sri Lanka (19), one from Caucasus (17), and three from Lebanon (16). The majority of homozygous mutations were found within the DHH domain of *PRUNE_1*, including c.G88A (p.D30N) in six patients from Oman (10) and one from Saudi Arabia (12), c.160C>A (p.P54T) in seven Iranian subjects (10), c.383G>A (p.R128Q) in two patients from Saudi Arabia (15), and c.515T>C (p.L172P) in three children from North Africa (16). Two types of homozygous mutations were also found within the DHHA2 domain: the missense variant c.C889T (p.R297W) in two patients from India (10) and the frameshift variant c.874_875insA (p.H292Qfs*3) in one subject from Turkey (52). Interestingly, different compound heterozygous mutations were also identified, comprising the c.[G383A];[G520T] [p.(R128Q); (G174X)] variant in two affected siblings from the USA (12) and two European patients (54) and c.[316G>A];[540T>A] [p.(D106N);(C180*)] in two Japanese subjects (17, 20). Furthermore, two different homozygous splicing variants were also described in several patients. In detail, a mutation in the splicing acceptor site (canonical) within intron 4 of *PRUNE_1* (c.521-2A>G [IVS4-2A>G]) was found in an Oji-Cree male (13) and in nine patients belonging to four Manitoba-Cree families (51). A different mutation in the splice donor site within intron 2 of the *PRUNE_1* gene (i.e., c.132+2T>C) was also described in five patients from two consanguineous Sudanese families (50). Moreover, homozygous deletion (g.1509-84457–151016-662del) starting from exon 2 to exon 8 including the 3′UTR was also found in two female children from Austria (16). Similarly, a homozygous truncating c.50dup (p.Leu18Serfs*8) variant was found in a Japanese child (17). Recently, a start loss c.3G>A (p.Met1)? variant in the *PRUNE_1* gene was also reported in two Iranian patients (53).

**Table 1:** List of genotyping features of patients affected by neurodevelopmental disorders (NDDs) due to mutations in *PRUNE_1* locus.

Nucleotide variations	Amino acid variations	Zygosity	Percentage of patients	No. of patients	Ethnicity	References
c.50dup	p.Leu18Serfs*8	Homozygous	1.56	1	Japan	(17)
c.G88A	p.D30N	Homozygous	10.94	6	Oman	(10)
				1	Saudi Arabia	(12)
c.132+2T>C	–	Homozygous	7.81	5	Sudan	(50)
c.160C>A	p.P54T	Homozygous	10.94	7	Iran	(10)
c.G316A	p.D106N	Homozygous	23.44	7	Turkey	(12, 16)
				3	Italy	(10)
				1	Sri Lanka	(19)
				1	Caucasus	(17)
				3	Lebanon	(16)
c.383G>A	p.R128Q	Homozygous	3.13	2	Saudi Arabia	(15)
c.515T>C	p.L172P	Homozygous	4.69	3	North Africa	(16)
c.521-2A>G	–	Homozygous	15.63	1	Oji-Cree	(13)
				9	Manitoba-Cree	(51)
540T>A	C180*	Homozygous	1.56	1	Japan	(17)
c.874_875insA	p.H292Qfs*3	Homozygous	1.56	1	Turkey	(52)
c.C889T	p.R297W	Homozygous	3.13	2	India	(10)
c.3G>A	M1?	Homozygous	3.13	2	Iran	(53)
g.1509-84457–151016-662del	–	Homozygous	3.13	2	Austria	(16)
c.[316G>A];[540T>A]	[p.(D106N);(C180*)]	Composite heterozygous	3.13	2	Japan	(17)
c.[G383A];[G520T]	[p.(R128Q);(G174X)]	Composite heterozygous	6.25	2	Europe	(54)
				2	USA	(12)

A genotype/phenotype correlation study was performed to dissect the function of mutant Prune_1 proteins focusing on the cG88A (p.D30N) and c.C889T (p.R297W) variants (10). Of interest is that the recombinant p.D30N and p.R297W mutant proteins (as synthetized and purified in *Escherichia coli*) were shown to have an increased PPX/PPase activity *in vitro* on tetraphosphates (P4) as substrates in comparison to the wild-type protein (*K*_cat_/*K*_m_ values: wild-type, 0.014 mM/s; p.D30N, 0.312 mM/s; p.R297W, 0.064 mM/s), thus suggesting a “gain-of-function” activity. The same mutant Prune_1 proteins (i.e., p.D30N and p.R297W) were also shown to affect both the cell proliferation and migration rates in NBL inducible cell clones (SH-SY5Y) (10). Furthermore, the MT polymerization kinetics was found delayed in the presence of the same mutant Prune_1 proteins both *in vitro* and in cells (10). Altogether, these *in vitro* results suggest that the cG88A (p.D30N) and c.C889T (p.R297W) variants in the *PRUNE_1* gene are gain-of-function mutations responsible for the alteration of cell proliferation and migration *via* impairment of MT polymerization using a neuronal model (i.e., SH-SY5Y cells). These results are in agreement with the homodimeric form of Prune_1 as reported *in vitro* (38) and confirmed in cells (41).

In contrast, another study reported that the missense c.G316A (p.D106N) and c.G383A (p.R128Q) variants result in a complete loss of PPX/PPase enzymatic activity on both P3 and P4 as substrates with respect to the wild-type Prune_1 protein, thus suggesting a loss-of-function activity for these mutants (54). Additionally, in the same paper, the authors showed an increased proteasome-dependent intracellular degradation of mutant p.D30N and p.D106N Prune_1 proteins using unsynchronized human embryonic kidney 293 (HEK-293) cells. In contrast, cells expressing the mutant p.G174∗ protein showed absence of Prune_1, while those expressing mutant p.R128Q resulted in an amount of Prune_1 protein comparable to that expressed by wild-type HEK-293 cells (54). Furthermore, homozygous null alleles in Prune_1 knockout mice were embryonic lethal at E9.5 with several vascular anomalies, including those in the yolk sac and in the cephalic vascular system (54). Thus, in contrast to the previous study, these data suggest a reduced function of mutant Prune_1 proteins due to hypomorphic variant alleles. Future research studies should be focused on this topic to functionally dissect these “gain or loss” single amino acid changes in *PRUNE_1* locus using *in vivo* state-of-the-art mouse development technology and/or functional analyses in fibroblast-derived organoids from affected patients.

## Prune_1 in Tumorigenesis

To date, high expressions of Prune_1 have been found in several metastatic solid tumors: MB groups 3 and 4 (11), gastric cancer (GC) (55), esophageal squamous cell carcinoma (ESCC) (56), NSCLC (8), thyroid cancer (TC) (57), colorectal cancer (CRC) (58), NBL (37), BC, and metastatic triple-negative breast cancer (TNBC) (6, 45, 59). Prune_1 overexpression occurs *via* the amplification and/or gain of chromosome 1q (as identified in tumors of epithelial origin), where *PRUNE_1* is located (i.e., 1q21.3) (6). For instance, MB group 3 (γ-subtype), in which Prune_1 was found overexpressed, has a trend for gain of chromosome 1q (60). Moreover, the gain of chromosomal region 1q21 was predominantly reported in BC belonging to the “basal-like” and TNBC subgroups (30%–40%) (61) and also in those recurrent (70%) (62), in which the levels of Prune_1 were found higher (45).

## Medulloblastoma

MB is an embryonal tumor occurring in the cerebellum and represents ∼20% of all primary childhood CNS tumors. Recent integrative genomics, messenger RNA (mRNA) expression, and methylation profiling have allowed MB to be stratified into different molecular subgroups: Wingless (WNT), Sonic Hedgehog (SHH), group 3, and group 4 (60). Heterogeneity within these subgroups has been recognized, and it has been suggested that MB may consist of up 12 subtypes [i.e., WNT (α and β), SHH (α, β, γ, and δ), group 3 (α, β, and γ), and group 4 (α, β, and γ)] (60). Distinctive transcriptional, mutational, and epigenetic profiles were reported for each MB subtype, with different clinical features (60). The MB SHH subgroup is characterized by an aberrant activation of the SHH pathway and arises from cerebellar granule neuron progenitors (GNPs) mainly mutated in PTCH1 or SMO receptors, although a non-canonical SHH/GLI activation has also been frequently observed (60). Although ∼60% of MB belongs to groups 3 and 4, future studies are needed to clarify the developmental origins and the biological mechanisms of these latest subgroups (60). MB group 3 is considered the most aggressive subgroup because of the high metastatic potential and the poor survival rate. The amplification of c-MYC is a common genetic feature in MB group 3 patients that was found to be inversely correlated with the clinical outcomes (63). One-third of MB group 4 patients are diagnosed with metastasis and show recurrent alterations in the genes involved in chromatin modification (64). Furthermore, amplifications of the *MYCN* and cyclin-dependent kinase 6 (*CDK6*) genes are alterations more commonly found in MB group 4 (65). In contrast, isochromosome 17q is found in more than 50% of both MB group 3 and 4 patients, thus being considered a common cytogenetic hallmark for these two subgroups. Of interest is that bulk high-throughput genomic profiling studies have recently reported a strong heterogeneity in MB groups 3 and 4 in terms of molecular and clinical features, thus showing a subset of tumors with overlapping signatures (66). The impact of this heterogeneity on therapy has been limited to trials testing Smoothened (SMO) antagonists for patients with MB SHH (67) and efforts to reduce therapy for those children affected by the MB WNT subgroup (68), who have relatively good outcomes. Outside of these trials, tailored therapies for MB groups 3 and 4 are currently lacking, and the combination of surgery, craniospinal radiotherapy (except in young children for whom radiotherapy has devastating neurocognitive side effects), and multi-agent chemotherapy is used (69).

Recently, high expressions of Prune_1 were found in these metastatic MB subgroups (i.e., MB groups 3 and 4), and a new metastatic axis (independent of c-MYC amplification) was dissected in MB group 3 with the poorest prognosis. In this regard, Prune_1 protein, due to the binding to Nm23-H1, enhances the canonical TGF-β pathway, thus leading to OTX2 and SNAIL upregulation, PTEN reduction, and EMT activation (11). Furthermore, gene expression and gene ontology analyses allowed other genes (i.e., *OTX2*, *CYFIP1*, and *GLI2*) involved in neurogenesis to be correlated to Prune_1 (11). Interestingly, a cell competitive permeable peptide (cell-penetrating peptide, CPP) that impairs Prune_1/Nn23-H1 complex formation was found to impair both tumorigenesis and metastatic spread in orthotopic models implanted with metastatic MB group 3 cells overexpressing c-MYC and mutated for TP53 (i.e., D425-Med cells), thus representing the most aggressive type of MB with the poorest prognosis (70). Furthermore, an anti-Prune_1 molecule (pyrimido-pyrimidine derivative, i.e., AA7.1) (23) was also found with the ability to bind Prune_1 protein, to enhance its intracellular degradation and to increase the PTEN protein level, thus inhibiting the Prune_1-mediated metastatic network both *in vitro* in primary human medullospheres obtained from patients and also *in vivo* in orthotopic xenograft models of metastatic MB group 3 (11).

### Other Tumours

#### Gastric Cancer

Prune_1 was found overexpressed in one-third of a cohort of human GC as measured *via* quantitative PCR (qPCR) and immunohistochemical analyses (55). Prune_1 was positively correlated with advanced tumor grade, lymph node metastasis, and poor prognosis. In this regard, GC patients expressing Prune_1 showed worse survival rates compared to those in which Prune_1 was not detected. Furthermore, the majority of these GC specimens (87%) were also found with positivity for the Nm23-H1 protein. Interestingly, the presence of Nm23-H1 was detected in all Prune_1-positive GC samples. Altogether, these findings indicate that the overexpression of both Prune_1 and Nm23-H1 is associated with poor clinical outcomes in GC (55).

#### Esophageal Squamous Cell Carcinoma

Prune_1 was also found increased in 21% of a cohort (*n* = 205) of ESCC patients. Its protein levels were found to be positively correlated with the tumor size (T; *p* < 0.0001) and regional lymph nodes (N; *p* < 0.0001). These data show that *PRUNE_1* could be used to identify ESCC patients with increased risk of disease recurrence or poor clinical outcomes (56).

#### Non-Small Cell Lung Cancer

Prune_1 was shown to modulate the Wnt pathway in NSCLC *via* its binding to GSK-3β and, as a consequence, the activation of β-catenin cascade (8). In this regard, Prune_1 silencing (through adenoviral approach) resulted in the reduction of activated β-catenin, inhibition of cell invasion, and decrease of the proliferation rate in NSCLC cells (8). Furthermore, the silencing of Prune_1 was also reported to inhibit lung metastasis formation *in vivo* using a murine xenograft model injected (*via* tail vein) with A549-Luc cells (overexpressing the firefly luciferase gene) silenced for Prune_1. Tumorigenesis was followed using *in vivo* bioluminescence (BLI) technology (71). Mice receiving these cells silenced for Prune_1 showed significant reduction of the tumor burden and lung metastasis nodules compared to those in the control group (8). Furthermore, Prune_1 protein was also detected in the serum of NSCLC patients *via* ELISA (8), thus suggesting that it could represent an early diagnostic marker for this type of tumors.

#### Thyroid Cancer

The role of Prune_1 in TC was also studied (57). In this regard, Prune_1 was detected in anaplastic TC and metastatic lymph node specimens. Prune_1 silencing and the inhibition of its PDE activity *via* dipyridamole were found to inhibit cell motility in human TC cells (i.e., 8505C and KTC-3) (57). These data were also confirmed *in vivo*, in which the inhibition of Prune_1 was reported to suppress tumor invasion and pulmonary metastasis in an orthotopic mouse model of TC (57). Thus, these analyses altogether confirm Prune_1 as a candidate target in anaplastic TC.

#### Colorectal Cancer

Prune_1 expression was found to be correlated with cell motility and EMT in CRC liver metastases (CRLM) (58). Prune_1 was found to positively regulate migration and EMT processes through *in vitro* assays performed using cells overexpressing or silenced for Prune_1 (58). Furthermore, *in vivo* experiments (with murine xenograft models) also confirmed the association of Prune_1 with tumor invasion and distant metastases (58). Interestingly, immunohistochemical analyses detected the Prune_1 protein in 28% of a cohort of CRC patients. Importantly, these Prune_1-positive CRC tumors showed significant lower overall survival (OS) rates (*p* = 0.003) (58).

#### Hepatocellular Carcinoma

Prune_1 was found overexpressed in HCC in a cohort of 304 patients using tissue microarray (TMA) (72). Of importance is that the expression of Prune_1 was found to be correlated with poorer OS and disease-free survival (DFS). Furthermore, gene expression analyses performed in HCC patients overexpressing Prune_1 demonstrated a statistically significant enrichment of the genes with roles in proliferative processes, DNA methylation, and the canonical Wnt pathway (72). Moreover, mutational spectra in HCC tumors with higher Prune_1 protein levels showed higher mutation burdens in *RPS6KA3* and *RB1* genes (72). Altogether, these findings suggest that *PRUNE_1* acts as a tumor promoter gene in HCC. Furthermore, at this time, its role in the epigenetic changes occurring in cancer-related genes can be postulated.

#### Breast Cancer and Triple-Negative Breast Cancer

High expression levels of Prune_1 were found associated with metastasis in regional lymph nodes (*p* = 0.017) and in distant sites (*p* = 0.029) in a cohort of BC-affected patients (45). These results were also recently confirmed in patients with TNBC through TMA immunohistochemistry technologies (59). In detail, the levels of Prune_1 protein had a positive correlation with distant metastasis (lung) and infiltrating pro-tumorigenic tumor-associated macrophages (M2-TAMs) with anti-inflammatory functions in the tumor microenvironment (TME). Furthermore, a genetically engineered mouse model (GEMM) of metastatic TNBC characterized by the overexpression of both human Prune_1 and Wnt1 in breast [through the use of the mouse mammary tumour virus (MMTV) promoter, i.e., MMTV-Prune_1/Wnt1] revealed that Prune_1 enhances the polarization of TAMs toward the M2 phenotype in the TME *via* the activation of the canonical (SMAD2-mediated) TGF-β pathway, IL17F secretion, and extracellular vesicle protein content modulation (59). Worth noting is that the non-toxic small anti-Prune_1 molecule (i.e., AA7.1) was found with the ability to impair the extracellular crosstalk between TNBC cells characterized by Prune_1 overexpression and TAMs, thus reducing the metastatic properties of the tumorigenic cells *in vivo* by using a GEMM of metastatic TNBC (i.e., MMTV-Prune_1/Wnt1) (59).

#### Neuroblastoma

Prune_1 and its interactor Nm23-H1 were also found significantly overexpressed in NBL (37). Interestingly, a positive correlation trend between Nm23-H1, Prune_1, and patients’ survival rates was also reported (37). Taken altogether, these findings suggest that the formation of the Prune_1/Nm23-H1 complex may have a role in cancer progression in NBL. Furthermore, high levels of Prune_1 and Nm23-H1 increased the aggressiveness of NBL cells (SH-SY5Y and SK-N-BE) *in vitro* and *in vivo* (37). In this regard, Prune_1 and Nm23-H1 were found to enhance the formation of metastatic foci in a mouse orthotopic xenograft model of NBL (37). Importantly, a cell CPP mimicking the region of Nm23-H1 that is responsible for its interaction with Prune_1 was found with the ability to impair the cell motility *in vitro* and the tumor growth and metastasis formation *in vivo* (37). Thus, the Prune_1/Nm23-H1 complex was found to enhance NBL tumorigenesis, and its impairment using CPP may be a useful strategy for NBL treatment.

## Prune_1 as Target for Anti-Tumorigenic Therapies

### Targeting Prune_1/Nm23-H1 Complex

Due to their prominent role in metastatic tumors, targeting the Prune_1/Nm23-H1 protein complex represents a promising therapeutic target for cancer treatment. The protein complex formation between Prune_1 and Nm23-H1 is dependent on casein kinase 1 (CKI)-mediated phosphorylation in S120, S122, and S125 on the C-terminus domain of Nm23-H1 protein (41). Thus, therapeutic strategies aimed to inhibit Prune_1/Nm23-H1 complex formation *via* targeting these phosphorylation processes could be applied in tumorigenic cells. In this regard, a CPP was developed to mimic the region of Nm23-H1 (from amino acids 115 to 129) that contains these residues (i.e., S120, S122, and S125) that are phosphorylated by the CKI enzyme. The ability of CPP to impair the Prune_1/Nm23-H1 complex was assayed in different tumorigenic cells *in vitro* (**Table 2**) (73). As a consequence, CPP reduced the cell proliferation and cell motility *in vitro* in BC, prostate cancer (PC), CRC, NBL, and MB cells (11, 73). The delivery of CPP in these tumorigenic cells was performed through adenoviral particles carrying the sequences encoding for CPP and for the transactivating protein of HIV. The absence of cytotoxicity *in vitro* in non-tumorigenic cells (i.e., HEK-293) indicated a specific action of CPP against cancer cells. The same adenoviral approach was also used *in vivo* (using xenograft murine models) to show the therapeutic benefits of CPP against PC, NBL, and MB (**Table 2**) (11, 37, 73). Of importance is that the biosafety of CPP in mice was also reported in terms of hematological parameters. Altogether, these data address the potential future use of CPP for the treatment of PC, NBL, and MB.

**Table 2:** List of the therapeutic strategies to impair Prune_1 *in vitro* and/or *in vivo*.

Tumour type	Treatment strategy	*In vitro* (cell lines)	*In vivo* (murine models)	References
Medulloblastoma (MB)	AA7.1 CPP	AA7.1: MB group 3 cell lines (D283-MED, D341-MED, D425-MED) and primary MB group3/4 cells CPP: MB group 3 cell lines (D283-MED and D425-MED)	AA7.1 and CPP: orthotopic xenograft mouse models with metastatic MB group 3 cell line (D425-MED)	(11)
Neuroblastoma (NBL)	CPP	NBL cell line (SH-SY5Y)	Mouse orthotopic xenograft models with NBL cells (SH-SY5Y)	(8, 73)
Triple-negative breast cancer (TNBC)	AA7.1 Dipyridamole CPP	AA7.1: murine TNBC cells (MMTV–Prune_1/Wnt1) Dipyridamole: murine and human TNBC cell lines (4T1 and MDA-MB-231T) CPP: TNBC cell lines (MDA-MB-231T)	AA7.1: GEMM of metastatic TNBC (MMTV–Prune_1/Wnt1 cells) Dipyridamole: orthotopic xenograft mouse models with TNBC cell lines (MDA-MB-231T and 4T1)	(22, 59, 73)
Breast cancer (BC)	CPP	BC (ER+, PG+) cell line (MCF-7)		(73)
Thyroid cancer (TC)	Dipyridamole		Orthotopic xenograft mouse models with TC cell line (8505C)	(57)
Prostate cancer	CPP	Prostate cancer cell line (PC3)	Orthotopic xenograft mouse models with prostate cancer cell line (PC3)	(73)
Colorectal cancer (CRC)	CPP	CRC cell lines (HT29 and SW480)		(73)

GEMM, genetically engineered mouse model; CPP, cell-penetrating peptide.

### Targeting Prune_1 Enzymatic Activities

The impairment of the PDE or PPX/PPase activities of Prune_1 represents another promising anti-tumorigenic strategy to impair tumor progression. In this regard, the PDE activity of Prune_1 was found to stimulate cellular motility *in vitro* and, as a consequence, metastatic progression. Furthermore, the anti-tumorigenic action of dipyridamole (an anti-platelet aggregation agent and one of the selective PDE inhibitors) in TNBC was also investigated *in vivo* using xenograft mice (**Table 2**) (22). Thus, PDE inhibitors could be used as therapeutic agents for Prune_1-overexpressing tumors. Interestingly, dipyridamole did not affect the PPX/PPase enzymatic function of Prune_1, as measured using P3 as a substrate (7).

The PPX/PPAse activity was shown to be diminished by Nm23-H1 *in vitro* (7). Indeed, Nm23-H1 was able to inhibit the P3-hydrolyzing activity of Prune_1 at micromolar concentrations (7). These data suggest a potential binding competition between Nm23-H1 and the P3 substrate for Prune_1. Thus, small-molecule activators of Nm23-H1 (e.g., NMac1) (74) represent future strategies to impair cancer progression in tumors characterized by the overexpression of Prune_1 and Nm23-H1.

It is of interest that long-chain polyPs (poor substrates of Prune_1) were also found to reduce the Prune_1-catalyzed hydrolysis of P3 substrate (7). This inhibitory effect may be due to Mg^2+^ chelation or to the competition between P3 and long-chain polyPs for active site binding of Prune_1. To date, other studies have reported on the anti-tumorigenic actions of polyPs. In this regard, the anti-metastatic activity of polyPs (P75) was reported for melanoma *in vivo* using murine xenograft models (75). Furthermore, treatment with polyPs (P120) was found to reduce the intracellular ATP level in NSCLC and increased their sensitivity to X-irradiation (76). Thus, polyPs can be promising targets for the development of novel anti-tumorigenic therapies in humans.

### Targeting Prune_1 as Downregulating Its Protein Levels

To date, a pyrimido-pyrimidine derivative (AA7.1) was found with the ability to decrease Prune_1 protein intracellular levels may be *via* enhancement of its proteasomal-dependent degradation (11). Protein–drug interaction studies (*via* NMR approaches) showed the amino acid residues of Prune_1 that are mainly responsible for the binding to AA7.1 (i.e., L359 and D364) (11). This molecule was also found able to decrease Prune_1 mRNA and protein levels in different MB group 3 (11) and TNBC (59) cells (**Table 2**). *In vitro* and *in vivo* assays also showed the ability of AA7.1 to decrease the cell proliferative and migratory processes (**Table 2**). In detail, in MB group 3, AA7.1 impaired the metastatic axis driven by Prune_1, thus leading to impairment in TGF-β, decreased levels of OTX2, upregulation of PTEN, inhibition of EMT, reduction in Nestin, and increases in Tuj1 and GFAP differentiation neuronal markers (11). These results suggest the potential of AA7.1 to inhibit the neural stem/progenitor cell markers and to increase neuronal differentiation processes, thus inhibiting the role of Prune_1 in these very important biological processes during neurogenesis.

Furthermore, the pharmacological inhibition of Prune_1 protein in TNBC (achieved through AA7.1 treatment) decreased the number of metastatic foci *in vivo* also *via* inhibiting the switch of TAMs in the TME toward the M2 phenotype (59). In TNBC, AA7.1-mediated tumor inhibition occurred through the impairment of the TGF-β pathway, reduction of inflammatory cytokines (i.e., IL-17F) and the modulation of the protein content of extracellular vesicles (i.e., vimentin) (59). Importantly, this small molecule is not toxic, as suggested by the lack of acute toxicity measured in naive mice (Balb/C) that were intraperitoneally administered escalating doses (15, 30, and 60 mg/kg) of AA7.1 daily for 1 week (11). The results showed no immediate acute toxicity of AA7.1 (in terms of hematological parameters, hepatotoxicity, or nephrotoxicity) in treated mice, as measured *via* glutamate–pyruvate transaminase 1, glutamic oxaloacetic transaminase, creatinine blood levels, and blood urea nitrogen (11). Thus, altogether, these findings suggest that AA7.1 is a non-toxic potential immunomodulatory molecule with the ability to modulate the inflammatory processes in the TME, thus inhibiting the metastatic spread in Prune_1-overexpressing tumors.

To date, none of these therapeutic approaches (as summarized in **Table 2**) has entered clinical testing. However, the pharmacological approach *via* Prune_1 downregulation (though AA7.1 treatment) has shown therapeutic benefits in murine models of MB and TNBC with the absence of acute toxicity (11). Regarding the inhibition of Prune_1/Nm23-H1 complex formation, CPP was also tested *in vivo* in xenograft mice of NBL and prostate cancer, thus also representing a promising strategy against tumors overexpressing both Prune_1 and Nm23-H1. Therefore, future studies should be focused on developing i) small molecules (e.g., AA7.1 derivatives) with a higher affinity to Prune_1 in order to reduce the dose to be used *in vivo* and ii) synthetic peptides with the ability to impair the interaction between Prune_1 and Nm23-H1, hence overcoming the use of adenoviral infection that might suffer from tissue-specific trophism.

## Discussion

Neural development involves a dynamic orchestrated sequence of cellular events driven by proliferation and migration processes involving neural progenitor cells (77). A tight spatial/temporal regulation of the proliferation and migration events in neural progenitor cells (NPCs) may prevent developmental malformations (as a result of hypo-proliferation) and tumor formation (due to hyper-proliferation). In this regard, NDDs (e.g., MCPH) may be caused by genetic mutations affecting the genes that modulate the proliferation and migration properties of NPCs. Similarly, brain tumorigenic cells can also derive from NPCs. Thus, the proliferation rate of brain tumorigenic cells may be regulated by the same genes that exert control on normal proliferative cells (78). Indeed, the different MB subgroups can originate from neural stem cells residing in the VZ of the cerebellum or from NPCs localized in the external granular layer of the cerebellum or rhombic lip (79). Since MB cells have been found to retain the genetic and molecular features of the cell of origin (80), the proteins involved in the growth and survival processes of neural stem cells or NPCs may also exert the same functions in brain tumorigenic cells.

Among these proteins, the *PRUNE_1* gene was reported both mutated in NDDs (i.e., NMIHBA) and overexpressed in pediatric brain tumors (i.e., MB). In this regard, Prune_1 was found with a fundamental role in the proliferation and migration processes occurring during neural development. Its expression, together with Nm23-H1, was detected in the mouse developing brain in the cortex, hippocampus, midbrain, and in the VZ, in which proliferation processes take place (5). Furthermore, mutations in the different regions of the human *PRUNE_1* gene (mainly in the region encoding for the DHH domain) were found to be causative of NDDs characterized by MCPH (the majority of the patients) and other brain anomalies (i.e., NMIHBA). At the same time, the Prune_1 protein was also described as overexpressed in both CNS and PNS tumors, including pediatric MB group 3, which drives the metastatic spread (11).

Thus, the deregulation of Prune_1 in somatic or germinal cells may result in oncogenesis or neural developmental defects, respectively. The explanation for this dual phenotypic effect might be provided by the pleiotropic role of Prune_1 in modulating several cellular processes, including proliferation, migration, MT polymerization during mitosis, and the regulation of signaling pathways.

We think that all these different functions are modulated by the enzymatic activities of Prune_1 (i.e., PPX/PPase and phosphodiesterase) and its property of being a naturally unfolded protein able to interact with other proteins with a role in these processes (i.e., Nm-23-H1/H2 and GSK-3β) (**Figure 1C**). How these interactions influence these processes is still to be discovered in detail in the near future. All these different functions are regulated by the enzymatic activities of Prune_1 (i.e., PPX/PPase and phosphodiesterase) and its unfolded structure in the C-terminus domain that allows it to interact with different proteins (i.e., Nm-23-H1/H2 and GSK-3β) (**Figure 1C**).

**Figure 2:**
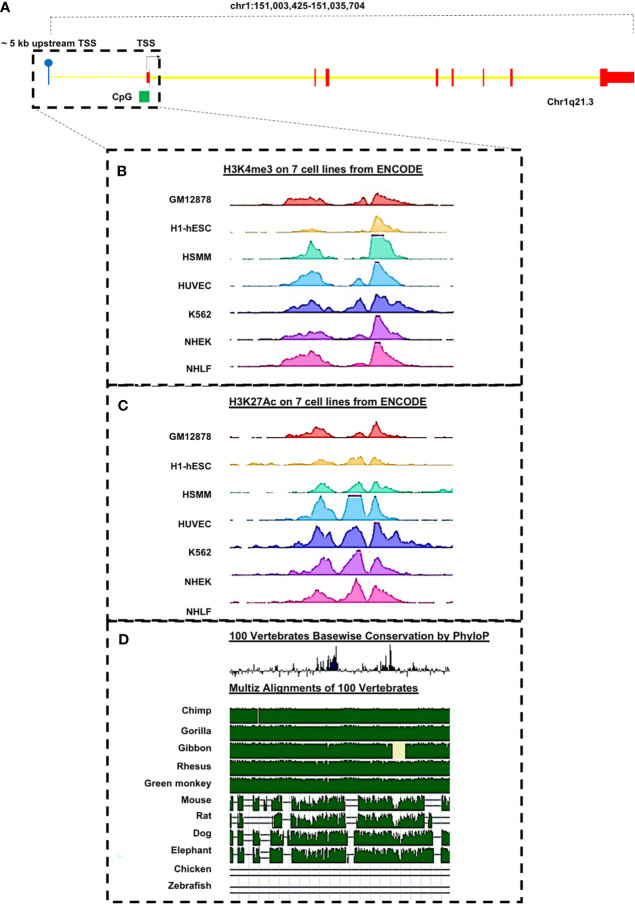
Representation of putative transcriptional factors predicted to bind the regulatory regions of the *PRUNE_1* gene. **(A)** Schematic representation of *PRUNE_1* gene and its upstream putative regulatory region as reported in the UCSC Genome Browser on Human Dec. 2013 (GRCh38/hg38) Assembly (https://genome.ucsc.edu/). The first exon (in *red*) is composed of a 5′UTR and a coding region. Introns are depicted in *yellow*. A CpG island (in *green*) is found in the promoter region of the gene. **(B)** The upstream region of *PRUNE_1* shows H3K4me3 peaks, as marker of the promoter region. **(C)** The upstream region of *PRUNE_1* shows H3K27Ac peaks, as marker of the enhancer regions. **(B, C)** Data obtained from chromatin immunoprecipitation sequencing (ChIP-seq) experiments performed on seven different cell lines, as reported in ENCODE (https://www.encodeproject.org/). **(D)** Peaks of the conserved genomic elements upon species are present in the upstream region of the gene.

Of interest is that, through *in silico* analyses of 5,000 bp upstream of the transcription start site (TSS) of the *PRUNE_1* gene, a number of sequences predicted to act as binding sites for transcriptional regulators have been identified (see **Figures 2A–D** and **Table 3**). This genomic region also contains sites (highly conserved through evolution) targeted by epigenetic modifications. Furthermore, the presence of a CpG island was also found (**Figure 2A**), thus suggesting potential DNA methylation sites associated with transcriptional repression. Overall, these predicted sites could be of importance for potential negative/positive modulation of *PRUNE_1* expression. Of note is that, among these putative transcriptional factors present at the 5′ region of the *PRUNE_1* gene (**Table 3**), we found mutations in *SMARCA4* that are available in a significant frequency of MB and pancreatic cancers and in many other tumor subtypes (81–83). *SMARCA4* was reported as the most frequently mutated gene in MB group 3 (83). Future research issues will be aimed at defining this finding and the transcriptional regulation of the *PRUNE_1* gene program in neurogenesis, development, epidermal differentiation, and cell cycle regulation. However, the roles and the transcriptional mechanisms of Prune_1 in both oncogenesis and NDDs still need further future investigations.

**Table 3:** List of putative transcription factor predicted to bind the regulatory elements (ORegAnno https://www.bcgsc.ca/resources/software/oreganno) 5000 bp upstream of the transcription start site of *PRUNE_1* gene.

Transcription factor	Chromosomal position (hg38 assembly)	Consensus sequences	Biological process
*CTCF*	chr1:151006584–151007134; chr1:151006744–151007094	5′-CCGCGNGGNGGCAG-3′	Development
*FOXA1*	chr1:151007690-151008630	5′-[AC]A[AT]T[AG]TT[GT][AG][CT]T[CT]-3′	Development
*RBL2*	chr1:151006386–151006759; chr1:151007954–151008902; chr1:151007961–151008350	Co-repressor (not directly binding to DNA)	Cell division/neuronal differentiation
*SMARCA4*	chr1:151004300–151009699	SWI/SNF subunit (chromatin remodeling complex)	Neuronal differentiation
*TFAP2C*	chr1:151007930–151008730	5′-GCCNNNGGC-3′	Neural tube development

**Figure 3:**
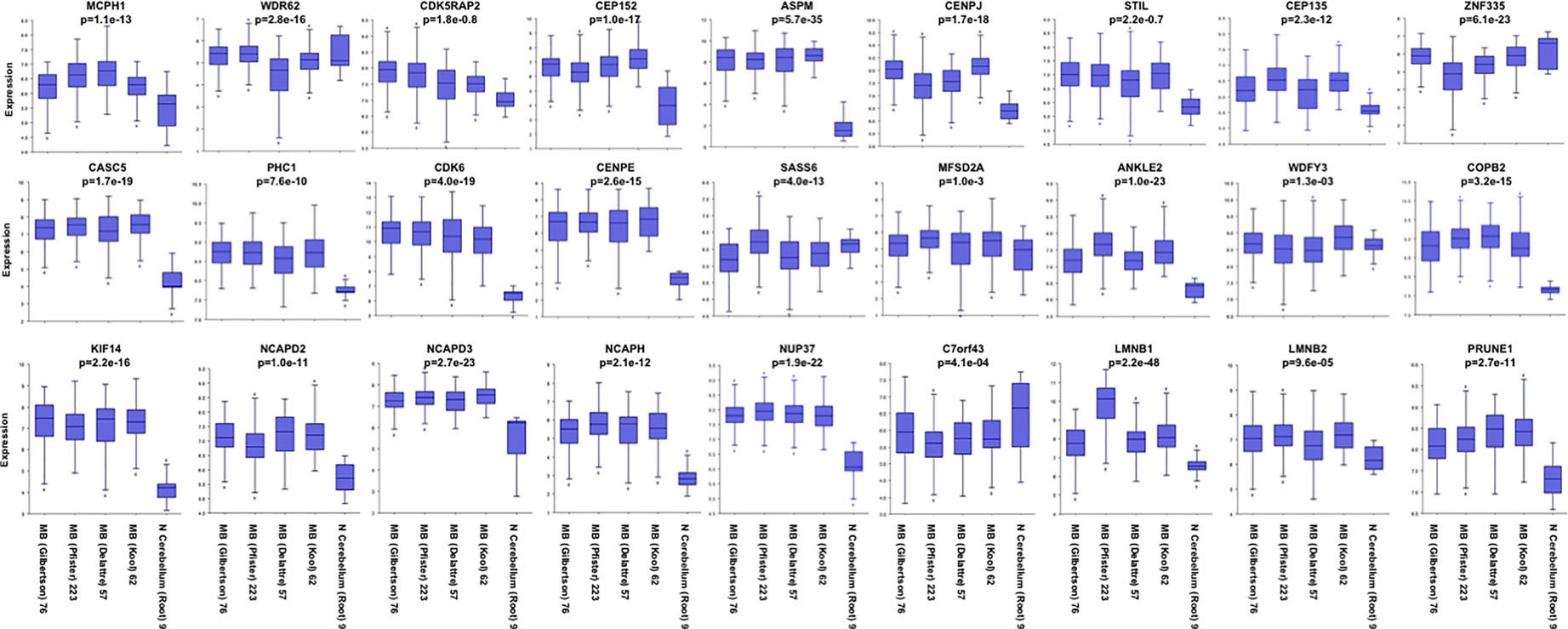
Representative expression gene panel list of genes that have been found causative of microcephaly (MCPH). RNA log2 expression of the genes reported as causative of primary MCPH (*MCPH1*, *WDR62*, *CDK5RAP2*, *CEP152*, *ASPM*, *CENPJ*, *STIL*, *CEP135*, *ZNF335*, *CASC5*, *PHC1*, *CDK6*, *CENPE*, *SASS6*, *MFSD2A*, *ANKLE2*, *WDFY3*, *COPB2*, *KIF14*, *NCAPD2*, *NCAPD3*, *NCAPH*, *NUP37*, *C7orf43*, *LMNB1*, and *LMNB2*) and *PRUNE_1* derived from transcriptome analysis of the primary cohorts of medulloblastoma (MB) in public available datasets (Pfister, Delattre, Gilbertson, and Kool) and normal cerebellum (Root) using the R2 Genomics Analysis and Visualization Platform (http://r2.amc.nl).

**Figure 4:**
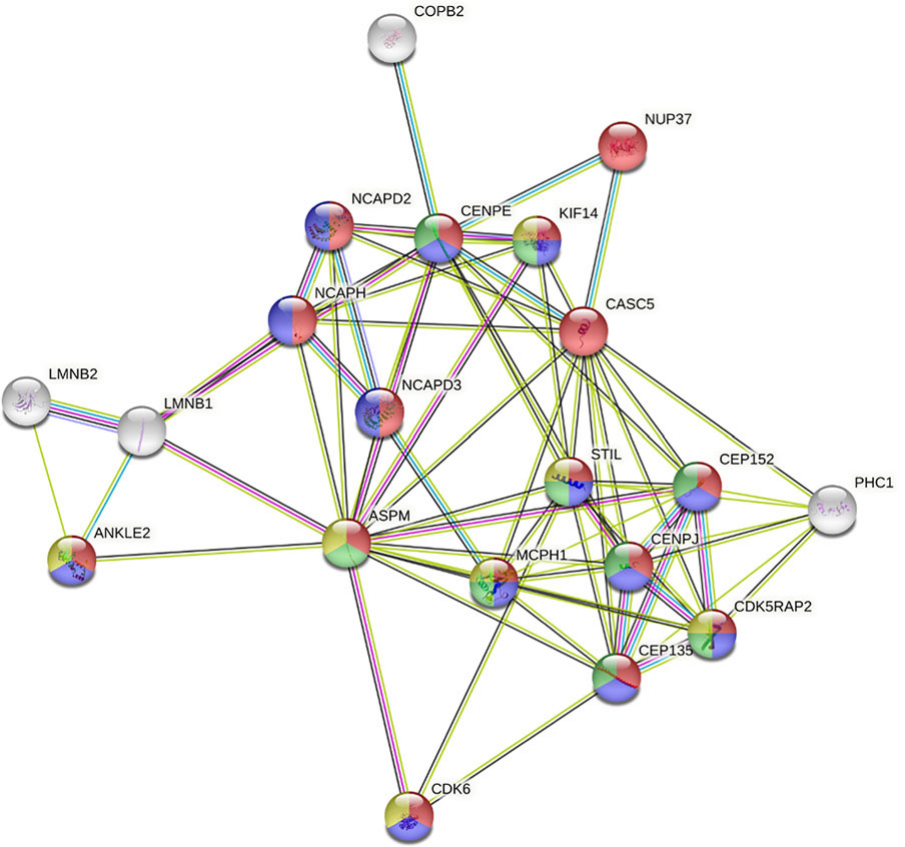
Protein network generated *via* STRING (Search Tool for Retrieval of Interacting Genes/Proteins). Analyses for the STRING database used the genes reported as causative of primary microcephaly (MCPH) and found overexpressed in medulloblastoma (MB) publicly available datasets (i.e., *MCPH1*, *CDK5RAP2*, *CEP152*, *ASPM*, *CENPJ*, *STIL*, *CEP135*, *CASC5*, *PHC1*, *CDK6*, *CENPE*, *ANKLE2*, *COPB2*, *KIF14*, *NCAPD2*, *NCAPD3*, *NCAPH*, *NUP37*, *LMNB1*, and *LMNB2*). The network is characterized by proteins involved in cell cycle (*red*, GO:0007049), mitotic cell cycle process (*blue*, GO:1903047), microtubule (MT)-based process (*green*, GO:0007017), and central nervous system (CNS) development (*yellow*, GO:0007417).

The mechanisms responsible for correct mitosis are of importance for both normal brain development and primary brain tumor growth (i.e., MB). Besides *PRUNE_1*, the other five genes (i.e., *KIF11*, *KIF14*, *ASPM*, *CDK6*, and *ATR*) have been reported as mutated in hereditary MCPH and overexpressed in primary brain tumors (e.g., MB). The products encoded by these genes are known to play crucial roles in regulating mitotic entry or cell cycle progression (78). Here, we analyzed public gene expression data related to the genes reported as causative of primary MCPH (47, 48) (**Table 4**) and *PRUNE_1* by interrogating available MB datasets (i.e., Pfister, Delattre, Gilbertson, and Kool) through the R2 Genomics Analysis and Visualization Platform (http://r2.amc.nl) (**Figure 3**). Interestingly, these data showed that the expression levels of the majority of these genes (i.e., 77.8%) were higher in MB samples compared to those in normal cerebellum (**Figure 3**). On these genes mutated in MCPH and overexpressed in MB, further analyses were performed using KEGG in order to identify a potential network of proteins (**Figure 4**). The data showed that these proteins are mainly involved in biological processes such as cell cycle (red, GO:0007049), mitotic cell cycle (blue, GO:1903047), MT-based process (green, GO:0007017), and CNS development (yellow, GO:0007417). Future studies will be needed to study the transcriptional activation of these genes and their functions on proliferation and migration in brain tumors.

**Table 4:** List of the genes identified as causative for primary microcephaly (MCPH).

Gene name	Gene ontology
*MCPH1*	Microcephalin. Implicated in chromosome condensation and DNA damage-induced cellular responses. May play a role in neurogenesis and regulation of the size of the cerebral cortex.
*CDK5RAP2*	CDK5 regulatory subunit-associated protein 2. Potential regulator of CDK5 activity *via* its interaction with CDK5R1. Negative regulator of centriole disengagement (licensing), which maintains centriole engagement and cohesion. Involved in the regulation of mitotic spindle orientation. Plays a role in spindle checkpoint activation by acting as a transcriptional regulator of both BUBR1 and MAD2 promoters. Together with MAPRE1, it may promote microtubule polymerization, bundle formation, growth, and dynamics at the plus ends. Regulates centrosomal maturation.
*CEP152*	Centrosomal protein of 152 kDa. Necessary for centrosome duplication; the function seems to also involve *CEP63*, *CDK5RAP2*, and *WDR62* through a stepwise assembled complex at the centrosome that recruits CDK2 required for centriole duplication. Acts as a molecular scaffold facilitating the interaction of *PLK4* and *CENPJ*, two molecules involved in centriole formation. Proposed to snatch *PLK4* away from *PLK4*:*CEP92* complexes in early G1 daughter centriole and to reposition *PLK4* at the outer boundary of a newly forming *CEP152* ring structure. Also plays a key role in deuterosome-mediated centriole.
*ASPM*	Abnormal spindle-like microcephaly-associated protein. Involved in mitotic spindle regulation and coordination of mitotic processes. Its function in regulating microtubule dynamics at spindle poles, including spindle orientation, astral microtubule density, and poleward microtubule flux, seems to depend on the association with the katanin complex formed by *KATNA1* and *KATNB1*. Enhances the microtubule lattice severing activity of *KATNA1* by recruiting the katanin complex to microtubules. Can block microtubule minus-end growth; reversely, this function can be enhanced by the katanin complex.
*CENPJ*	Centromere protein J. Plays an important role in cell division and centrosome function by participating in centriole duplication. Inhibits microtubule nucleation from the centrosome. Involved in the regulation of slow processive growth of centriolar microtubules. Acts as a microtubule plus-end tracking protein that stabilizes centriolar microtubules and inhibits microtubule polymerization and extension from the distal ends of centrioles. Required for centriole elongation and for *STIL*-mediated centriole amplification. May be involved in the control of centriolar microtubule growth.
*STIL*	SCL-interrupting locus protein. Immediate-early gene. Plays an important role in embryonic development and in cellular growth and proliferation. Its long-term silencing affects cell survival and cell cycle distribution and decreases CDK1 activity correlated with the reduced phosphorylation of CDK1. Plays a role as a positive regulator of the Sonic Hedgehog pathway, acting downstream of *PTCH1*. Plays an important role in the regulation of centriole duplication. Required for the onset of procentriole formation and proper mitotic progression.
*CEP135*	Centrosomal protein of 135 kDa. Centrosomal protein involved in centriole biogenesis. Acts as a scaffolding protein during early centriole biogenesis. Required for the targeting of centriole satellite proteins to centrosomes such as of *PCM1*, *SSX2IP*, and *CEP290* and the recruitment of *WRAP73* to centrioles. Also required for centriole–centriole cohesion during interphase by acting as a platform protein for *CEP250* at the centriole. Belongs to the *CEP135*/*TSGA10* family.
*CASC5*	Kinetochore scaffold 1. Performs two crucial functions during mitosis: it is essential for spindle assembly checkpoint signaling and for correct chromosome alignment. Required for the attachment of the kinetochores to the spindle microtubules. Directly links *BUB1* and *BUB1B* to kinetochores. Part of the MIS12 complex, which may be fundamental for kinetochore formation and proper chromosome segregation during mitosis. Acts in coordination with *CENPK* to recruit the NDC80 complex to the outer kinetochore.
*PHC1*	Polyhomeotic-like protein 1. Component of a Polycomb group (PcG) multiprotein PRC1-like complex, a complex class required to maintain the transcriptionally repressive state of many genes, including *Hox* genes, throughout development. PcG PRC1 complex acts *via* chromatin remodeling and modification of histones. It mediates the monoubiquitination of histone H2A “Lys-119,” rendering chromatin heritably changed in its expressibility. Required for proper control of cellular levels of *GMNN* expression.
*CDK6*	Cyclin-dependent kinase 6. Serine/threonine protein kinase involved in the control of cell cycle and differentiation. Promotes G1/S transition. Phosphorylates pRB/RB1 and NPM1. Interacts with D-type G1 cyclins during interphase at G1 to form a pRB/RB1 kinase and controls the entrance into the cell cycle. Involved in the initiation and maintenance of cell cycle exit during cell differentiation. Prevents cell proliferation and negatively regulates cell differentiation, but is required for the proliferation of specific cell types (e.g., erythroid and hematopoietic cells).
*CENPE*	Centromere-associated protein E. Microtubule plus-end-directed kinetochore motor that plays an important role in chromosome congression, microtubule–kinetochore conjugation, and spindle assembly checkpoint activation. Drives chromosome congression (alignment of chromosomes at the spindle equator resulting in the formation of the metaphase plate) by mediating the lateral sliding of polar chromosomes along spindle microtubules toward the spindle equator and by aiding the establishment and maintenance of connections between kinetochores and spindle microtubules.
*ANKLE2*	Ankyrin repeat and LEM domain-containing protein 2. Involved in mitotic nuclear envelope reassembly by promoting the dephosphorylation of BAF/BANF1 during mitotic exit. Coordinates the control of BAF/BANF1 dephosphorylation by inhibiting VRK1 kinase and promoting the dephosphorylation of BAF/BANF1 by protein phosphatase 2A (PP2A), thereby facilitating nuclear envelope assembly. It is unclear whether it acts as a real PP2A regulatory subunit or whether it is involved in the recruitment of the PP2A complex. Involved in brain development.
*COPB2*	Coatomer subunit beta. The coatomer is a cytosolic protein complex that binds to dilysine motifs and reversibly associates with Golgi non-clathrin-coated vesicles, which further mediate biosynthetic protein transport from the ER *via* the Golgi up to the trans-Golgi network. The coatomer complex is required for budding from Golgi membranes and is essential for the retrograde Golgi-to-ER transport of dilysine-tagged proteins. In mammals, the coatomer can only be recruited by membranes associated with ADP-ribosylation factors (ARFs), which are small GTP-binding proteins.
*KIF14*	Kinesin-like protein KIF14. Microtubule motor protein that binds to microtubules with high affinity through each tubulin heterodimer and has an ATPase activity. Plays a role in many processes such as cell division, cytokinesis, and also in cell proliferation and apoptosis. During cytokinesis, targets to the central spindle and midbody through its interaction with *PRC1* and *CIT*, respectively. Regulates cell growth through the regulation of cell cycle progression and cytokinesis. During cell cycle progression, acts through SCF-dependent proteasomal ubiquitin-dependent protein.
*NCAPD2*	Condensin complex subunit 1. Regulatory subunit of the condensin complex, a complex required for the conversion of interphase chromatin into mitotic-like condense chromosomes. The condensin complex probably introduces positive supercoils into relaxed DNA in the presence of type I topoisomerases and converts nicked DNA into positive knotted forms in the presence of type II topoisomerases. May target the condensin complex to DNA *via* its C-terminal domain. Belongs to the CND1 (condensin subunit 1) family.
*NCAPD3*	Condensin-2 complex subunit D3. Regulatory subunit of the condensin-2 complex, a complex that establishes mitotic chromosome architecture and is involved in the physical rigidity of the chromatid axis.
*NCAPH*	Condensin complex subunit 2. Regulatory subunit of the condensin complex, a complex required for the conversion of interphase chromatin into mitotic-like condense chromosomes. The condensin complex probably introduces positive supercoils into relaxed DNA in the presence of type I topoisomerases and converts nicked DNA into positive knotted forms in the presence of type II topoisomerases.
*NUP37*	Nucleoporin Nup37. Component of the Nup107-160 subcomplex of the nuclear pore complex (NPC). The Nup107-160 subcomplex is required for the assembly of a functional NPC. The Nup107-160 subcomplex is also required for normal kinetochore microtubule attachment, mitotic progression, and chromosome segregation.
*LMNB1*	Lamin-B1. Lamins are components of the nuclear lamina, a fibrous layer on the nucleoplasmic side of the inner nuclear membrane, which is thought to provide a framework for the nuclear envelope and may also interact with chromatin.
*LMNB2*	Lamin-B2. Lamins are components of the nuclear lamina, a fibrous layer on the nucleoplasmic side of the inner nuclear membrane, which is thought to provide a framework for the nuclear envelope and may also interact with chromatin. Belongs to the intermediate filament family.
*WDR62*	WD repeat-containing protein 62. Required for cerebral cortical development. Plays a role in neuronal proliferation and migration. Plays a role in mother-centriole-dependent centriole duplication. The function seems to also involve *CEP152*, *CDK5RAP2*, and *CEP63* through a stepwise assembled complex at the centrosome that recruits CDK2 required for centriole duplication.
*MFSD2A*	Sodium-dependent lysophosphatidylcholine symporter 1. Sodium-dependent lysophosphatidylcholine (LPC) symporter, which plays an essential role for blood–brain barrier formation and function. Specifically expressed in the endothelium of the blood–brain barrier of microvessels and transports LPC into the brain. Transport of LPC is essential because it constitutes the major mechanism by which docosahexaenoic acid (DHA), an omega-3 fatty acid that is essential for normal brain growth and cognitive function, enters the brain.
*SASS6*	Spindle assembly abnormal protein 6 homolog. Central scaffolding component of the centrioles ensuring their ninefold symmetry. Required for centrosome biogenesis and duplication: required both for mother-centriole-dependent centriole duplication and deuterosome-dependent centriole amplification in multi-ciliated cells. Overexpression results in excess foci-bearing centriolar markers. Required for the recruitment of *STIL* to the procentriole and for *STIL*-mediated centriole amplification.
*WDFY3*	WD repeat and FYVE domain-containing protein 3. Required for selective macroautophagy (aggrephagy). Acts as an adapter protein by linking specific proteins destined for degradation to the core autophagic machinery members, such as the ATG5-ATG12-ATG16L E3-like ligase, SQSTM1, and LC3. Along with p62/SQSTM1, involved in the formation and autophagic degradation of cytoplasmic ubiquitin-containing inclusions (p62 bodies, ALIS/aggresome-like induced structures). Along with SQSTM1, required to recruit ubiquitinated proteins to PML bodies in the nucleus. Important for normal brain development.
*ZNF335*	Zinc finger protein 335. Component or associated component of some histone methyltransferase complexes that may regulate transcription through the recruitment of these complexes on gene promoters. Enhances ligand-dependent transcriptional activation by nuclear hormone receptors. Plays an important role in neural progenitor cell proliferation and self-renewal through the regulation of specific genes involved in brain development, including *REST*. Also controls the expressions of genes involved in somatic development and regulates, for instance, lymphoblast proliferation.
*C7ORF43*	Chromosome 7 open reading frame 43, with unknown function.

Intriguingly, Prune_1 was reported to activate the canonical Wnt pathway through its binding to GSK-3β. However, its overexpression in MB was found in metastatic groups 3 and 4, but not in tumors belonging to the Wnt molecular subgroup. This may be due to the different frequencies of genetic aberration (e.g., amplification of chromosome 1q) in the different subtypes of MB (60) and also because of the heterogeneity among the MB subtypes and their distinct developmental origins. In this regard, the cerebellum is thought to be the origin site for MB SHH, group 3, and group 4, while the lower rhombic lip of the developing brain stem seems to represent the site from which MB WNT arises (79). More in detail, the cells of origin for MB SHH are the granule cell precursors (GCPs) (79). In contrast, MB group 3 develops from Nestin**^+^** cerebellar stem cells, and MB group 4 derives from progenitor cells giving rise to both GCP and unipolar brush cell (UBC) lineages (79).

To date, among the mutations identified in *PRUNE_1* locus in NMIHBA patients, only three gene variants have been reported within the public data of Catalog of Somatic Mutations in Cancer (COSMIC, v94, released May 29, 2021) of 39,615 samples collected. In detail, the missense p.D30N Prune_1 variant (COSM5843581) was found in one patient affected with malignant melanoma, the p.R297W mutation (COSM462922) was reported in one subject suffering from clear cell renal cell carcinoma and in three patients diagnosed with colon adenocarcinoma, and the splicing c.521-2A>G variant (COSM7836151) was found in one patient with squamous cell lung carcinoma. Nevertheless, because of the paucity of tumor samples with *PRUNE_1* mutations and due to the lack of information regarding the zygosity of the identified variants, we cannot hypothesize a role for these *PRUNE_1* mutations in tumorigenic processes. Thus, further studies will be needed to address this issue.

Different genotype to phenotype correlation studies have been reported to dissect the role of mutant Prune_1 protein in children affected with NMIHBA. In this regard, opposing enzymatic activities for mutated Prune_1 proteins have been reported (10, 54). The contrasting results about the biochemical activity of the mutant Prune_1 proteins could be reasoned by the homodimeric form reported for Prune_1 (38), the interaction with intracellular binding partners (whose protein abundance is dependent of the cell district) that can modulate its enzymatic function (e.g., Nm23-H1) (7), and the possible oscillatory changes in Prune_1 protein abundance (and activity) during the cell cycle because of its role in the mitotic spindle during mitosis (10). Thus, further studies taking into account the potential role of polyPs at short chain lengths (as Prune_1 substrates) in neurogenesis and the homodimeric structure of Prune_1 and its interactors will be needed to address this issue.

Of interest is that Prune_1 acts as a MAP, enhances the MT polymerization (*in vitro* and in cells), and co-localizes with β-tubulin in the mitotic spindle in dividing cells (10). Importantly, its interactor, Nm23-H1, was also described to be an active constituent of the centrosomes (84) and was reported to be associated with α- and β-tubulins with a role in their polymerization (85, 86), probably acting as a source of GTP necessary for α- and β-tubulins during their assembly. Altogether, these data suggest a potential role for the Prune_1 and Nm23-H1 complex during MT polymerization processes.

**Figure 5:**
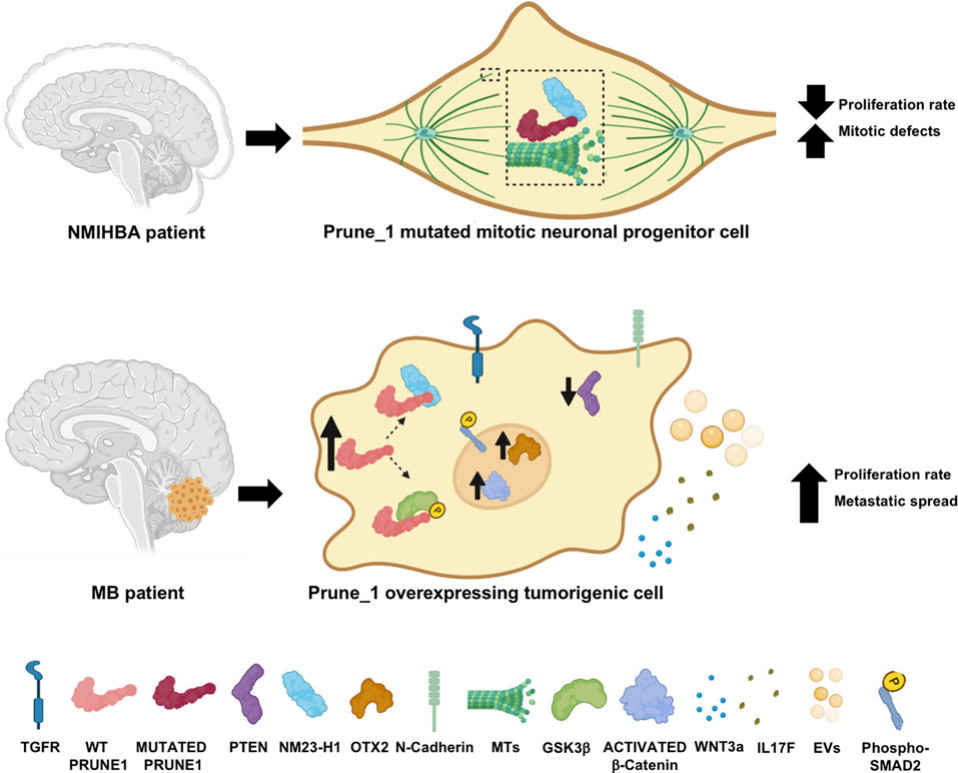
Cartoon representation illustrating the hypothesis of the mechanisms of action of Prune_1 when overexpressed in medulloblastoma (MB) tumorigenic cells (*upper panel*) or mutated in neural progenitor cells of patients with neurodevelopmental disorder with microcephaly, hypotonia, and variable brain anomalies (NMIHBA) (*bottom panel*). *Upper panel*: Prune_1 acting as a microtubule-associated protein (MAP) in the mitotic spindle thus increases the microtubule (MT) polymerization rate. Similarly, Nm23-H1 was also shown to take part in polymerization processes. When Prune_1 is mutated (e.g., p.D30N and p.R297W), MT polymerization is delayed, thus unbalancing the MT dynamics in mitotic cells, causing mitotic defects and decreasing the cell proliferation rate. *Bottom panel*: Once overexpressed in tumorigenic cells (e.g., MB), Prune_1 was reported to enhance canonical TGF-β pathways *via* interaction with Nm23-H1, thus increasing phosphorylated SMAD2 and leading to OTX2 upregulation, PTEN inhibition, and epithelial–mesenchymal transition (EMT) activation (through the upregulation of N-cadherin). Prune_1 was also shown to enhance canonical Wnt signaling through its interaction with GSK-β, which causes the increase of its inhibitory phosphorylation on Ser9 and Ser21 residues, thus leading to the activation of β-catenin. Through the activation of these two signaling cascades, Prune_1 can also take part in extracellular pathways, thus increasing the secretion of Wnt3a and IL17F and modulating the protein contents of extracellular vesicles (EVs). These actions are responsible for the increased proliferation rate of tumorigenic cells and their metastatic dissemination.

At this time, we can postulate that the Prune_1 protein may be implicated in both NDD and brain tumor development mostly due to its enzymatic activities and its ability to activate signaling cascades (**Figure 5**). Prune_1, by acting as a MAP, enhances MT polymerization, thus modulating the dynamics of MTs in the mitotic spindle during mitosis. Thus, in NMIHBA patients, mutated Prune_1 proteins were found to be responsible for the delayed MT polymerization and the decreased cell proliferation and migration processes (**Figure 5**, upper panel). On the other hand, in tumorigenic cells, the Prune_1 protein was found to activate canonical Wnt (*via* its binding to GSK-3β), thus promoting the activation of β-catenin and the secretion of Wnt3a. Prune_1 was also reported to enhance the TGF-β pathway (through interaction with Nm23-H1), increase OTX2 and N-cadherin levels, and reduce PTEN levels. Moreover, Prune_1 was reported to modulate the secretion of soluble cytokines (including IL-17F) and the vesicle protein content (e.g., vimentin). These mechanisms of action lead to the increase of the cell proliferation rate of tumorigenic cells and their metastatic spread (**Figure 5**, bottom panel).

In conclusion, Prune_1 is emerging as a novel attractive target in both NDD and brain tumorigenesis. To date, several approaches (e.g., pharmacological inhibition or cell competitive permeable peptides) to impair Prune_1 protein, its enzymatic activities, or its interaction with other protein binding partners have been tested *in vitro* and/or *in vivo* in preclinical animal models (**Table 2**). Furthermore, the development of new small molecules able to rescue the altered enzymatic activity of mutated Prune_1 proteins could also be useful for the treatment of tumors in which the same mutations in *PRUNE_1* locus were found.

Despite the promising results here presented related to the use of novel modalities of Prune_1 inhibitors, these small molecules have not been tested yet in clinical settings. We envision, at this time, that aggressive and metastatic tumors will greatly benefit from these inhibitors in new clinical programs.

## Author Contributions

FB and CS performed the expression analyses and generated the protein network and the gene ontology. VF and MZ wrote the manuscript. All authors discussed the data and contributed to the final work.

## Funding

This study was supported by PRIN Ministero dell’Università e Ricerca Italiana Project no. 2017FNZRN3 (MZ), the Italian Association for Cancer Research (AIRC; grant IG no. 22129) to MZ, Fondazione Cariplo (to VF and MZ), Fondazione Celeghin Italiana (to MZ), and Ministero dell’Università e della Ricerca Italiana (PRIN; grant no. 2017FNZRN3) (to MZ).

## Conflict of Interest

The authors declare that the research was conducted in the absence of any commercial or financial relationships that could be construed as a potential conflict of interest.

## Publisher’s Note

All claims expressed in this article are solely those of the authors and do not necessarily represent those of their affiliated organizations, or those of the publisher, the editors and the reviewers. Any product that may be evaluated in this article, or claim that may be made by its manufacturer, is not guaranteed or endorsed by the publisher.
